# Integration and acceptability of cervical cancer screening as part of postnatal care among women attending postnatal clinics in the Accra Metropolis, Greater Accra Region, Ghana

**DOI:** 10.1371/journal.pgph.0004070

**Published:** 2024-12-31

**Authors:** Lawrence Oppong Buadi, Raphael Odame, Ali Samba, Mumuni Kareem, Angela Amoanimaa Boateng, Claudette Ahliba Diogo, Rebecca Tricia Morrision, George Kumi Kwakye, Cecilia Smith-Togobo

**Affiliations:** 1 Greater Accra Regional Hospital, Accra, Ghana; 2 Family Health Division, Ghana Health Service, Accra, Ghana; 3 Department of Obstetrics and Gynaecology, University of Ghana Medical School, Accra, Ghana; 4 School of Public Health, University of Port Harcourt, Port Harcourt, River State, Nigeria; 5 Department of Medical Laboratory Sciences, University of Health and Allied Sciences, Ho, Ghana; Universiti Malaya, MALAYSIA

## Abstract

Cervical cancer is a recognized preventable disease yet the fourth most common cancer among women globally. This study assessed the integration and acceptability of cervical cancer screening as part of routine sixth-week postnatal care among women attending a postnatal clinic. This was a cross-sectional descriptive study among 347 postpartum women who were attending their 6th-week postnatal visit. A Pap smear test was performed on each consenting study participant. Only conventional cervical smears were performed. The Pap smear samples were taken using a special kit (PAP-PAK Cytology brush kit) for a 3-smear sampling of the ectocervix (emphasis on the squamo-columnar junction), endocervix (endocervical canal past squamo-columnar junction) and the posterior fornix via a cervical-vaginal scraper and a CytoSoft cytology brush respectively. All smears were fixed with 95% ethyl alcohol and allowed to dry in cool air. The samples were sent to the cytology laboratory and stained with the Pap staining technique. All the smears were examined and reported by the cytology department of Korle-Bu Teaching Hospital. Descriptive statistics such as frequency tables were drawn and proportions were estimated. Bivariable analysis between categorical variables and outcome variables was done. A p-value ≤ 0.05 was considered statistically significant. Most (90.5%) of the study participants were satisfied with the Pap test procedure and about 52.7% indicated that paying Gh₵60.00 ($12.50) for screening test was affordable. The outcome of Pap smear tests revealed that most (90.8%) of the participants had normal Pap test results. In the univariable analysis, participants’ educational level (p = 0.006); occupation (p < 0.001), and contraceptive use (p = 0.019) were significantly associated with the acceptability of the Pap test procedure. The multivariable analysis revealed that educational level (aOR = 3.27; 95% CI = 1.05–10.21; p = 0.041) and occupation (aOR = 6.49; 95% CI = 1.67–25.29; p = 0.007) were significantly associated with the acceptability of the Pap test procedure and showed higher odds of acceptability. Integration of cervical cancer screening into the routine sixth-week postnatal clinic has the potential to be feasible with anticipated high uptake. We therefore recommend a pilot study be initiated to integrate cervical cancer screening into the routine maternal health services as part of postnatal care. Also, the Ministry of Health/Ghana Health Service should initiate discussion with the National Health Insurance Authority for possible inclusion as part of the national health insurance scheme’s benefit package in the nearby future.

## Introduction

Cervical cancer is a recognized preventable disease yet the fourth most common cancer among women globally [1]. According to the World Health Organisation (WHO), an estimated 604,000 new cases and 343,000 cervical cancer deaths were recorded in 2020 [1]. WHO further revealed that approximately 90% of all the new cases and death across the globe in 2020 occurred in low- and middle-income countries. Cervical cancer is largely caused by Human Papilloma Virus (HPV) infection which is acquired through sexual intercourse [2]. HPV is responsible for approximately 99% of cervical cancer, and almost all cervical pre-malignant lesions [3–5].

Cervical cancer is the most common gynaecological cancer in developing countries in Africa where most of the patients present late [6]. This presents a significant public health threat to women across the African continent. Despite the limited country-specific data, the International Agency for Research reveals that 111,632 new cases of cervical cancer were recorded in sub-Saharan Africa in 2018 of which in the same year, an estimated 68% of women died from cervical cancer [7]. Sub-Saharan Africa reports the highest regional incidence and mortality, where every year 34.8 per 100,000 women are diagnosed with cervical cancer and 22.5 per 100,000 women die from the disease [8–10].

The Catalan Institute of Oncology (ICO) and the International Agency for Research on Cancer (IARC) reports that a population of 10.6 million women aged 15 years and older are at risk of developing cervical cancer in Ghana [11]. Again, the Catalan Institute of Oncology (ICO) and the International Agency for Research on Cancer (IARC) rank cervical cancer as the second most common cancer among women in Ghana as well as the second most common cancer among women between 15 and 44 years [11]. It is estimated that 2797 women are diagnosed with cervical cancer every year while 1699 women die from the disease [11].

Even though cervical cancer is well known to be a preventable disease through vaccination, screening and treatment of premalignant lesions, these interventions are not effectively utilized in many low-income countries including Ghana [12]. For instance, there is no systematic national screening and treatment program for premalignant lesions of the cervix in Ghana. However, in urban areas where some cervical screening services may be available, they are at best opportunistic. Adanu et al in 2010 reported that cervical cancer screening is low even among adult women who visit hospitals in Accra [2]. The absence of a national and systematic screening program in Ghana puts a large proportion of women in their reproductive age at high risk of developing cervical cancer [13]. Cervical premalignant lesions are usually picked up by way of screening tests thereby preventing their later consequences. Pap smears can significantly minimize the risk of dying from cervical cancer. It has reduced worldwide deaths from cervical cancer by approximately 2% annually since its inception in 1941 [13]. It is also known to have reduced the overall mortality rate by 74% [13]. The accuracy of pap smears in pregnancy is similar to that of non-pregnant women [14]. Therefore, integrating cervical cancer screening using the Pap smear test into the postnatal care service will allow most of our women to be screened. The sixth week postnatal visit is often the last time that many young women encounter the health system for a long while and thus presents a good opportunity for screening tests in low-resource countries such as Ghana. This notwithstanding, there are currently no interventions to screen for cervical cancer among postnatal women as part of regular postnatal services using pap smear tests, and neither are there any systematic empirical studies in Ghana looking at the feasibility and acceptability of integrating Pap smear tests as part of routine sixth-week postnatal care among women attending a postnatal clinic. This gap could potentially hamper efforts to develop effective cervical cancer detection and treatment strategies. As evidence has shown, countries that have been involved in mass-organized cytology-based screening programs, have seen a decline in the incidence and mortality of cervical cancer [15]. It is for this reason that this study was conducted to fill the current gap in both literature and practice in Ghana. Therefore, the study aimed to determine the feasibility and acceptability of Pap smear tests as part of routine sixth-week postnatal care among women attending a postnatal clinic in selected hospitals in the Accra Metropolis.

## Methods

### Study design

This was a cross-sectional descriptive study among postpartum women who were attending their 6^th^-week postnatal visit at the postnatal clinics of the Trust Hospital, Accra Regional Hospital (Ridge Hospital), La General Hospital and Lekma Hospital in the Accra Metropolis and have consented to be part of the study. Data were collected on 347 women seeking care for their sixth week’s postnatal clinic at four selected hospitals in the Accra metropolis, and this was done from 1^st^ February to 30^th^ April, 2018 until data collection was completed and analysis done for the study.

### Inclusion criteria

- Postpartum women who were asymptomatic of cervical cancer.
- Postpartum women attending their sixth week postnatal clinic and consent to be part of the study

### Exclusion criteria

- Women with a history of vaginal douching in the preceding 48 hours of the screening
- Women with a history of vaginal medication in the preceding 48 hours of the screening
- Women with or are found to have invasive cervical cancer
- Women who had a cervical injury during labour that was repaired (as enquired from participants)
- Women whose routine Pap smear schedule is not due (since they are on a routine Pap test schedule) were excluded from the study.

### Sample size

The required sample size (N) was calculated using Cochrane’s sample size formula [16] for cross-sectional studies and with the following assumptions on the proportion of abnormal Pap smear tests among women at 30%, therefore with an estimated prevalence; p = 30% [17,18] at 95% confidence level and 5% margin of error, the minimum sample required for the study after 10% non-response rate and/or participants withdrawal was 355.

Study participants were recruited at their two weeks postnatal visit but the study was carried out when they turned up at their sixth-week postnatal clinic. The women were approached at the two weeks postnatal visit and subsequently invited to partake in the study after the study objectives, the Pap smear procedure and the prerequisites (viz. abstaining from douching, using antiseptic cream in the vagina and coitus for at least 48 hours before the day of the smear) were explained to them. Those who agreed to be part of the study had their folder numbers recorded for identification on the day of the smear, that is, their sixth-week postnatal clinic visit.

### Study variables

The variables under this study are the Pap smear test, acceptability of Pap smear test and affordability of Pap smear test as the dependent variable and the independent variables include reproductive and contraceptive history (age at menarche, age at first sexual intercourse, retroviral status, lifetime sexual partners, contraception use, contraception type); and socio-demographic characteristics (age, educational level, marital status, occupation, parity, gravidity and smoking).

### Data collection

In the sixth week postnatal clinic, the women who agreed to be part of the study were approached to ascertain whether they were still interested in partaking in the study. Those who still showed interest were screened as per the prerequisites for the Pap smear protocol and using the exclusion criteria for this study. Those who were found to have fulfilled the requirement were asked to provide a signed informed consent form and an interviewer-administered structured questionnaire containing both open and closed-ended questions was administered to each participant. Participants who could write and read self-administered the questionnaires while participants unable to read and write underwent interviewer-administered by the research assistants. Data was collected on the socio-demographic characteristics of the participant, personal and family history, and reasons for wanting to or not having a pap smear done. A Pap smear test was then performed on each consenting study participant. Only conventional cervical smears were performed. The Pap smear samples were taken using a special kit (PAP-PAK Cytology brush kit) for a 3-smear sampling of the ectocervix (emphasis on the squamo-columnar junction), endocervix (endocervical canal past squamo-columnar junction) and the posterior fornix via a cervical-vaginal scraper and a CytoSoft cytology brush respectively. All smears were fixed with 95% ethyl alcohol and allowed to dry in cool air. The samples were sent to the cytology laboratory and stained with the Pap staining technique. All the smears were examined and reported by the cytology department of Korle-Bu Teaching Hospital. All study participants were counselled before the Pap smear test results of the women were communicated to them via phone calls after receiving results from the cytology laboratory.

### Data management and statistical analysis

The data was entered into an Excel spreadsheet, cleaned and exported to Statistical Package for the Social Science (SPSS) version 23 for analysis. Descriptive statistics such as frequency tables were drawn and the proportions were estimated to determine the percentage of women who accepted to have a Pap smear done at the sixth week’s post-natal visit. The proportion of women at the sixth week’s postnatal who were eligible for a Pap smear as per the study’s protocol was calculated. The proportion of women at the sixth week’s postnatal clinic who had a satisfactory Pap smear was also determined. Univariable analysis between socio-demographic characteristics of women and acceptability of Pap test procedure was done to determine association and the corresponding p-values were calculated. Bivariable analysis between socio-demographic characteristics of the women and abnormal Pap smear test was done and the corresponding p-values were calculated. All the various proportions calculated and bivariable analysis in this study were done at a 95% confidence level.

### Ethical approval

The Ethical and Protocol Review Committee of the College of Health Sciences, University of Ghana approved this current study with Protocol Identification Number: CHS-Et/M.1–P 1.1/2017-2018. The Ghana College of Physicians and Surgeons’ Ethical Review Board also approved this study. Institutional permission to undertake the study was sought from the Regional Director of Health Services, Greater Accra Region and the heads of the four selected hospitals. All the study participants and service providers consented to the study.

## Results

### Socio-demographic characteristics of study participants

A total of 347 postpartum women were recruited for the study. A little above half (53.6%) of the study participants were between 30 and 39 years old. The mean age was 29.6 (±4.7) with ages 18 and 42 years as the minimum and maximum ages respectively. Most (78.4%) of the participants were married. A majority (60.5%) were in informal occupations. About two-thirds (66.2%) of participants had both secondary and tertiary education. Most (85.0%) had gravidity of less than 5, and almost all (98.3%) participants had given birth less than five times [Table 1].

**Table 1:** Socio-demographic characteristics of study participants.

Variable	Frequency (N = 347)	Percentage, % [Range]
**Mean age** (±SD) **Age (years)**	29.6 (±4.7)	[18 – 42]
< 20	6	1.7
20–29	150	43.2
30–39	186	53.6
≥ 40	5	1.4
**Marital Status**		
Single	72	20.7
Married	272	78.4
Divorced	3	0.9
**Educational level**		
None	33	9.5
Primary	84	24.2
Secondary	123	35.4
Tertiary	107	30.8
**Occupation**		
Formal	112	32.3
Informal	210	60.5
Unemployed	25	7.2
**Cigarette smoking**		
Yes	6	1.7
No	341	98.3
**Gravidity**		
< 5	295	85.0
≥ 5	52	15.0
**Parity**		
< 5	341	98.3
≥ 5	6	1.7

### Reproductive health and contraceptive history of study participants

Out of a total of 347 participants, a majority (63.4%) had their first menarche at the age of more than 14 years. The mean age was 15.0 (±1.9). About (79.8%) of the study participants had their first sexual intercourse at the age of 18 years and above, with a mean age of 20.3 (±3.5). Most (89.6%) were HIV-negative. Less than half (45.8%) had ever used contraception in the past with only (6.9%) currently using contraception.

On Pap smear test acceptability, affordability and test outcomes, most (90.5%) of the study participants were very satisfied with the pap test procedure and a little above half (52.7%) indicated that the cost of the test was affordable. The outcome of Pap smear tests revealed that most (90.8%) of the participants had normal Pap test results [Table 2].

**Table 2:** Reproductive health and contraceptive history of study participants.

Variable	Frequency (N = 347)	Percentage, % [Range]
**Age at menarche (years)**		
≤ 14	127	36.6
> 14	220	63.4
Mean age at menarche (±SD)	15.0 (±1.9)	[10 – 20]
**Age at first sexual intercourse (years)**		
< 18	70	20.2
≥ 18	277	79.8
Mean age at first sexual intercourse (±SD)	20.3 (±3.5)	[13 – 32]
**Retro (HIV) status**		
Positive	3	0.9
Negative	311	89.6
Not indicated	33	9.5
**Lifetime sexual partner**		
1	135	38.9
2	140	40.3
≥ 3	72	20.7
**Contraception history**		
Ever used	159	45.8
Never used	188	54.2
**Contraceptive use**		
Current use	24	6.9
Ever used	135	38.9
Never	188	54.2
**Contraceptive type (n = 159)**		
Condom	51	32.1
Depo	3	1.9
Implant	20	12.6
Inject	40	26.2
Intra-uterine contraceptive device	12	7.6
Oral contraceptive pill	24	15.1
Pill	3	1.9
Other	6	3.7
**Pap test acceptability at PNC**		
Yes	303	87.3
Uncertain	44	12.7
**Acceptability of pap test procedure**		
Very satisfied	314	90.5
Somehow satisfied	33	9.5
**Recommend pap test at PNC**		
Yes	316	91.1
Maybe	31	8.9
**Affordability of pap test cost (Gh₵60.00 / $12.50)**		
Affordable	183	52.7
Not affordable	164	47.3
**Pap smear test result**		
Abnormal test result	2	0.6
Normal pap test result	315	90.8
Unsatisfactory smear	30	8.6

Not indicated = missing numbers.

### Factors associated with acceptability of Pap test procedure

In the univariable analysis, participants’ educational level (p = 0.006); occupation (p < 0.001), and contraceptive use (p = 0.019) were significantly associated with the acceptability of the Pap test procedure [Table 3].

**Table 3:** Factors associated with the acceptability of Pap test procedure.

Factors	Acceptability		Chi-square/Fischer’s exact/ (p-value)
	Very Satisfied n (%)	Somehow satisfied n (%)	
**Age (years)**			0.728 ^a^
< 20	6 (100.0)	0 (0.0)	
20–29	138 (92.0)	12 (0.8)	
30–39	165 (88.7)	21 (11.3)	
≥ 40	5 (100.0)	0 (0.0)	
**Marital Status**			0.080^a^
Single	60 (83.3)	12 (16.7)	
Married	251 (92.3)	21 (7.7)	
Divorced	3 (100.0)	0 (0.0)	
**Educational level**			0.006^a^
None	33 (100.0)	0 (0.0)	
Primary	72 (85.7)	12 (14.3)	
Secondary	117 (95.1)	6 (4.9)	
Tertiary	92 (86.0)	15 (14.0)	
**Occupation**			18.4 (<0.001)
Formal	94 (83.9)	18 (16.1)	
Informal	201 (95.7)	9 (4.3)	
Unemployed	19 (76.0)	6 (24.0)	
**Cigarette smoking**			0.01 (0.965)
Yes	144 (90.6)	15 (9.4)	
No	170 (90.4)	18 (9.6)	
**Gravidity** ^a^			0.444
< 5	265 (89.8)	30 (10.2)	
≥ 5	49 (94.2)	3 (5.8)	
**Parity** ^a^			1.000
< 5	308 (90.3)	33 (9.7)	
≥ 5	6 (100.0)	0 (0.0)	
**Contraceptive use**			8.0 (0.019)
Current use	18 (75.0)	6 (25.0)	
Ever used	126 (93.3)	9 (6.7)	
Never	170 (90.4)	18 (9.6)	
**Affordability of pap test cost (Gh₵60.00 / $12.50)**			0.05 (0.827)
Affordable	165 (90.2)	18 (9.8)	
Not affordable	149 (90.9)	15 (9.1)	
**Pap smear test result**			0.174 ^a^
Abnormal test result	2 (100.0)	0 (0.0)	
Normal pap test result	282 (89.5)	33 (10.5)	
Unsatisfactory smear	30 (100.0)	0 (0.0)	

^a^Analyzed using Fischer’s exact.

Further, multivariable analysis also revealed that educational level and occupation are significantly associated with the acceptability of the Pap test procedure. The logistic regression analysis of the factors associated with the acceptability of the pap test procedure shows women that had secondary education were about a little above 3 times (AOR = 3.27; 95% CI = 1.05–10.21) more likely to accept pap test procedures compared with those that had primary education. Similarly, women that had informal occupations were almost 6.5 times (AOR = 6.49; 95% CI = 1.67–25.29) more likely to accept the Pap test procedure compared with those who had formal occupations [Table 4].

**Table 4:** Logistic regression analysis of the factors associated with the acceptability of the Pap test procedure.

Variables	Model 1		Model 2	
	Unadjusted OR (95%CI)	P–value	Adjusted OR (95%CI)	P–value
**Educational level**				
None	-		-	
Primary	1.00		1.00	
Secondary	3.25 (1.17–9.04)	0.024	3.27 (1.05–10.21)	0.041
Tertiary	1.02 (0.45–2.32)	0.958	3.48 (0.80–15.09)	0.096
**Occupation**				
Formal	1.00		1.00	
Informal	4.28 (1.85–9.87)	0.001	6.49 (1.67–25.29)	0.007
Unemployed	0.61 (0.21–1.73)	0.349	1.53 (0.32–7.41)	0.596
**Contraceptive use**				
Current use	1.00		1.00	
Ever used	4.67 (1.49–14.66)	0.008	2.17 (0.66–7.14)	0.203
Never	3.15 (1.11–8.94)	0.031	1.84 (0.61–5.61)	0.281

Note: In Model 2, we adjusted for all the variables that were significant in Model 1.

OR: Odds ratio.

CI: Confidence interval.

## Discussion

A larger number (87.3%) of the women who attended the postnatal clinics accepted cervical cancer screening using the conventional Pap smear test and therefore agreed to do it again if the opportunity avails itself at a postnatal visit. This finding was similar to a study done on knowledge and acceptability of Pap smear, self-sampling and HPV vaccination among adult women (though not strictly among postpartum women) in Kenya by Rositch and colleagues, where most of the women held positive attitudes towards future Pap smear screening [19]. The acceptability by a large number of the women may have been due to the education that was given on cervical cancer and the relevance of screening to identify premalignant lesions for early treatment and prevention of late diagnosis of the disease. It was noticed that most of the women did not even know what the Pap smear test is and its ability to prevent invasive cervical cancer. Therefore, increasing awareness and literacy of cervical cancer risk factors, treatment and prevention strategies will translate into an increase in the uptake of cervical cancer screening programmes across the country. This is also evident in a study by Ebu and colleagues on knowledge, practice and barriers towards cervical cancer screening in Elmina. Their study revealed that lack of knowledge on risk factors, treatment, and prevention strategies about cervical cancer i.e., Pap smear tests were among the factors that hinder women from patronizing cervical cancer screening services [13]. Further, in support of the call for increased awareness creation and cervical cancer literacy, Ndikom and colleagues also emphasized the urgent need for more awareness and education about cervical cancer especially by health workers [20]. Again, the literature revealed that women who had heard of HPV and knew that Pap smears are used to prevent invasive cervical cancer were far more likely to find Pap screening 100% acceptable and necessary as compared to women without the knowledge of HPV and the role of Pap smears in cervical cancer prevention [19]. Therefore, increasing awareness and knowledge of women and the general populace on cervical cancer would go a long way to enhancing positive attitudes towards cervical cancer screening and prevention.

In our current study, most (90.5%) of the women were very satisfied with the Pap smear test procedure and this translated to 91.1% of the women agreeing to recommend the six weeks postnatal Pap smear test to relations as compared to the smaller number (8.9%) who disagreed because they were uncomfortable with the procedure. The large response in terms of satisfaction with the Pap smear procedure by the women signifies that good training of service providers in performing Pap smear will also encourage women to access this screening service as some women shy away from Pap smear test because they deem it a painful procedure. Good training of service providers in performing Pap smear procedures makes the procedure less uncomfortable and/or painful. This finding agrees with a study carried out in El Salvador on factors affecting attendance to cervical cancer screening among women where the authors revealed that under-screened women reported painful procedures as one of the factors that prevented them from accessing cervical cancer screening services [21]. In the same study, some of the reasons mentioned for the low uptake of screening include but are not limited to lack of awareness, inadequate access and procedure discomfort [21]. Also, the reported 12.7% in our study that were not sure if they will undergo the screening test again gave reasons such as anxiety in waiting for results and the Pap smear test not being a comfortable procedure among others. Further confirmation of this finding is revealed in a study by Ebu and colleagues in Elmina, Southern Ghana on knowledge, practice and barriers toward cervical cancer screening [13]. In their study, the researchers reported that painful screening experiences inhibited participation [13]. In less developed countries, prohibitive rates of mortality due to cervical cancer persist as a result of a lack of effective screening programmes and low uptake of Pap smear testing [22]. Therefore, making the procedure less painful will motivate women to access Pap smear tests as a tool for the prevention of cervical cancer.

A larger number (91.4%) of the smears were satisfied thus making it possible to have a Pap smear test on a six-week postpartum cervix since by this time the pre-pregnancy state of the cervix would have been achieved and adequate sampling of the endocervical canal and transformation zone possible. In support of this, literature reveals that a study on the clinical parameters associated with the absence of endocervical/transformation zone components in conventional cervical Papanicolaou smears revealed an incidence of an unsatisfactory smear of 8.7%, which was similar to that seen in our study [23]. However, the researchers argued that the presence or absence of an endocervical/transformation zone was due to ageing, conization, pregnancy and the postpartum period. Therefore, postpartum women according to the researchers, are supposed to have more endocervical/transformation cells due to grossly ectropic cervix and no coating mucus as seen in pregnancy which facilitates endocervical/transformation zone cell collection [23]. Further, the average time spent by each participant for the Pap smear procedure was 5 minutes, which was less time spent (for the procedure), as an overwhelming number (90.5%) of the participants were very satisfied with the procedure thus supporting the integration of the screening programme with limited waiting time spent at the clinic. The Pap smear test kit and reading of results cost an average of sixty Ghana cedis approximately 12.50 dollars and all the women were willing to pay at least half the cost making it affordable for government to only subsidize and have such a great national programme initiated. Therefore, investing in cervical cancer screening programmes using Pap smear tests will reduce the socio-economic burden on the individual and the country at large [24]. Moreover, a hospital-based cross-sectional study on patient side cost and its predictors for cervical cancer in Ethiopia revealed that cervical cancer causes a huge financial burden on patients [25]. However, the study placed much emphasis on primary preventive measures such as vaccination against HPV and cervical cancer screening and recommended its induction and scaling up to reduce morbidity from cervical cancer and catastrophic expenditure incurred on clients and their families [25,26].

On the role of screening in postpartum period, literature reveals that cervical cancer screening during postpartum is key to increase coverage and uptake [27]. In a study by [27], the authors revealed that during pregnancy or after delivery a significant number of women in reproductive age visit a health facility in Ghana. Their study reported that 66.4% of postnatal women visited a health facility and were screened for cervical cancer [27]. In addition, a quality improvement study by [28] revealed that the low cervical cancer screening uptake in England saw improvement in uptake when screening services and information were targeted at postnatal women. This is because women in postpartum have multiple contact with health professionals which serve as an opportunity to promote cervical cancer screening and uptake [28]. This further highlights the vital role of screening in postpartum period thereby a good call to integrate cervical cancer screening in postpartum care to increase coverage and uptake in a low resource country like Ghana.

## Conclusion

In Ghana, some centres in the country provide a “screen and treat” strategy using VIA, as well as other centres that provide Pap smear tests as a cervical cancer screening method for detecting pre-malignant lesions of the cervix, however, most of these centres do opportunistic screening. A national programme where cervical cancer screening is integrated into the routine postnatal clinic where most of our women can be captured will therefore be welcomed and thus help in the prevention, early detection and treatment of cervical cancer among our women who usually report late for treatment.

Integration of cervical cancer screening into the routine sixth-week postnatal clinic is therefore potentially feasible as shown in this study, and we recommend that cervical cancer screening should be integrated into the routine maternal health services (as part of postnatal care). The Ministry of Health/Ghana Health Service should initiate discussion with the National Health Insurance Authority for possible inclusion as part of the national health insurance scheme’s benefit package in the nearby future to enhance affordability and accessibility. Also, postnatal clinics be equipped by the Ministry of Health and Ghana Health Service for the delivery of premalignant cervical lesion screening using the conventional Pap smear test since it is easy to train midwives to undertake such services. This will help prevent late presentation of cervical cancer among women and as well increase accessibility to screening services.

## Limitation of study

This is not a health economics study. Therefore, the study did not assess the cost component and cost effectiveness of integrating cervical cancer screening into routine sixth-week postnatal clinic instead it assessed the integration and acceptability of the screening test. Also, cervical cancer screening could also be done by HPV testing which is more sensitive to enhance detection rates of abnormalities, however, in a low resource country like Ghana this is not affordable yet but will replace Pap smears in the near future. This might also account for the low abnormal Pap smear test reported in this study.

## Supporting information

S1 Data (XLS)

## References

[1] World Health Organisation. Overview of Cervical Cancer (2023). cervical cancer (who. int) on 22^nd^ February 2023

[2] Adanu RM, Seffah JD, Duda R, Darko R, Hill A, Anarfi J. Clinic visits and cervical cancer screening in Accra. Ghana medical journal. 2010; 44(2). doi: 10.4314/gmj.v44i2.68885 21327005 PMC2994147

[3] Elamurugan S, Rajendran P, Thangamani S. Cervical cancer screening: Awareness, attitude, and practice of Indian women. Tropical Journal of Medical Research. 2016 Jan 1; 19(1):42-.

[4] Franceschi S. The IARC commitment to cancer prevention: the example of papillomavirus and cervical cancer. Tumour prevention and genetics III. 2005:277–97. doi: 10.1007/3-540-26980-0_18 15648196

[5] Banura C, Mirembe FM, Katahoire AR, Namujju PB, Mbonye AK, Wabwire FM. Epidemiology of HPV genotypes in Uganda and the role of the current preventive vaccines: A systematic review. Infectious agents and cancer. 2011 Dec; 6(1):1–2. 21749691 10.1186/1750-9378-6-11 PMC3163594

[6] Nkyekyer K. Pattern of gynaecological cancers in Ghana. East African medical journal. 2000; 77(10). doi: 10.4314/eamj.v77i10.46708 12862120

[7] World Health Organisation. Regional Office for Africa (2023). cervical cancer | WHO | Regional Office for Africa on 17th May, 2023

[8] Catarino R, Petignat P, Dongui G, Vassilakos P. Cervical cancer screening in developing countries at a crossroads: Emerging technologies and policy choices. World Journal of clinical oncology. 2015 Dec 12; 6(6):281. 26677441 10.5306/wjco.v6.i6.281 PMC4675913

[9] African Health Organisation. Cervical Cancer in Africa Fact Sheet (2020) https://aho.org/fact-sheets/cervical-cancer-in-africa-fact-sheet on 17th May 2023

[10] Ferlay J, Soerjomataram I, Dikshit R, Eser S, Mathers C, Rebelo M, Parkin DM, Forman D, Bray F. Cancer incidence and mortality worldwide: sources, methods and major patterns in GLOBOCAN 2012. International journal of cancer. 2015 Mar 1; 136(5): E359–86. doi: 10.1002/ijc.29210 25220842

[11] Catalan Institute of Oncology (ICO) and the International Agency for Research on Cancer (IARC) (2023). Information Centre on HPV and Cancer. Ghana: Human Papillomavirus and Related Cancers, Fact Sheet 2023 (hpvcentre.net) on 15^th^ May 2023

[12] Abdullah F, O’Rorke M, Murray L, Su TT. Evaluation of a worksite cervical screening initiative to increase Pap smear uptake in Malaysia: a cluster randomized controlled trial. BioMed research international. 2013 Jan 1; 2013. 10.1155/2013/572126 PMC3773923 24073411

[13] Ebu NI, Mupepi SC, Siakwa MP, Sampselle CM. Knowledge, practice, and barriers toward cervical cancer screening in Elmina, Southern Ghana. International journal of women’s health. 2014 Dec 24:31–9. doi: 10.2147/IJWH.S71797 25565902 PMC4284003

[14] Himabindu P, Kanwal A, PG V. Pap Smear in antenatal women-Routine screening in low resource settings. IOSR Journal of Dental and Medical Sciences. 2015; 14(4):4–5.

[15] Denny L. Cytological screening for cervical cancer prevention. Best Practice & Research Clinical Obstetrics & Gynaecology, 2012; 26(2), 189 196. doi: 10.1016/j.bpobgyn.2011.08.001 22071306

[16] Israel GD. Determining sample size. University of Florida. Fact Sheet PEOD-6 November 1992

[17] Maleki A, Ahmadnia E, Avazeh A, Mazloomzadeh S, Molaei B, Jalilvand A. Prevalence of abnormal papanicolaou test results and related factors among women living in Zanjan, Iran. Asian Pacific journal of cancer prevention. 2015; 16(16):6935–9. doi: 10.7314/apjcp.2015.16.16.6935 26514471

[18] Donkoh ET, Agyemang-Yeboah F, Asmah RH, Wiredu EK. Prevalence of cervical cancer and pre-cancerous lesions among unscreened Women in Kumasi, Ghana. Medicine. 2019 Mar; 98(13). doi: 10.1097/MD.0000000000014600 30921178 PMC6456016

[19] Rositch AF, Gatuguta A, Choi RY, Guthrie BL, Mackelprang RD, Bosire R, Manyara L, Kiarie JN, Smith JS, Farquhar C. Knowledge and acceptability of pap smears, self-sampling and HPV vaccination among adult women in Kenya. PloS one. 2012 Jul 10; 7(7): e40766. doi: 10.1371/journal.pone.0040766 22808257 PMC3393696

[20] Ndikom CM, Ofi BA. Awareness, perception and factors affecting utilization of cervical cancer screening services among women in Ibadan, Nigeria: a qualitative study. Reproductive health. 2012 Dec; 9:1–8. 22866676 10.1186/1742-4755-9-11 PMC3539913

[21] Alfaro KM, Gage JC, Rosenbaum AJ, Ditzian LR, Maza M, Scarinci IC, Miranda E, Villalta S, Felix JC, Castle PE, Cremer ML. Factors affecting attendance to cervical cancer screening among women in the Paracentral Region of El Salvador: a nested study within the CAPE HPV screening program. BMC Public Health. 2015 Dec; 15:1–8. 26474762 10.1186/s12889-015-2360-7 PMC4609068

[22] Urasa M, Darj E. Knowledge of cervical cancer and screening practices of nurses at a regional hospital in Tanzania. African health sciences. 2011; 11(1). PMC3092321 21572857

[23] Sun L, Wang PH, Lee CH, Fu TF, Chou MM, Hwang SF, Ke YM, Hsu ST, Lu CH. Clinical parameters associated with absence of endocervical/transformation zone component in conventional cervical Papanicolaou smears. Taiwanese Journal of Obstetrics and Gynecology. 2016 Feb 1; 55(1):81–4. doi: 10.1016/j.tjog.2014.03.015 26927255

[24] Randall TC, Ghebre R. Challenges in prevention and care delivery for women with cervical cancer in sub-Saharan Africa. Frontiers in oncology. 2016 Jun 28; 6:160. doi: 10.3389/fonc.2016.00160 27446806 PMC4923066

[25] Hailu A, Mariam DH. Patient side cost and its predictors for cervical cancer in Ethiopia: a cross-sectional hospital-based study. BMC cancer. 2013 Dec; 13:1–8. 23391288 10.1186/1471-2407-13-69 PMC3576296

[26] World Health Organisation. Global strategy to accelerate the elimination of cervical cancer as a public health problem 2020; file:///C:/Users/toshiba/Downloads/9789240014107-eng.pdf

[27] Effah K, Tekpor E, Klutsey GB, Bannor HT, Amuah JE, Wormenor CM, Kemawor S, Danyo S, Atuguba BH, Manu LS, Essel NO. Antenatal and postnatal cervical precancer screening to increase coverage: experience from Battor, Ghana. Ecancermedicalscience. 2023; 17. doi: 10.3332/ecancer.2023.1616 38414944 PMC10898892

[28] Coleridge SL, Wiggans A, Nelissen E, Bethune R, Blackwell R, Bryant A, Morrison J. Improving the uptake of cervical screening in pregnant and recently postnatal women: a quality improvement project. BMJ Open Quality. 2022 May 1; 11(2):e001709. doi: 10.1136/bmjoq-2021-001709 35613829 PMC9134207

